# Potential Benefits of Therapeutic Use of ****β**2**-Adrenergic Receptor Agonists in Neuroprotection and Parkinson**μ**s Disease

**DOI:** 10.1155/2014/103780

**Published:** 2014-01-19

**Authors:** Lynda Peterson, Kathleen P. Ismond, Elisha Chapman, Patrick Flood

**Affiliations:** ^1^Theralogics, Inc., 1829 E. Franklin Street, Chapel Hill, NC 27514-5861, USA; ^2^School of Dentistry, University of Alberta, 7020 Katz Group Centre, Edmonton, AB, Canada T6G 2R3; ^3^North Carolina Oral Health Institute, School of Dentistry, The University of North Carolina at Chapel Hill, Manning Dr. & Columbia Street, Campus Box 7450, Chapel Hill, NC 27599-7450, USA

## Abstract

The **β**2-adrenergic receptor (**β**2AR) is a seven-transmembrane (7TM) G-protein coupled receptor that is expressed on cells of the pulmonary, cardiac, skeletal muscle, and immune systems. Previous work has shown that stimulation of this receptor on immune cells has profound effects on the regulatory activity of both adaptive and innate immune cells. This review examines the functional dichotomy associated with stimulation of **β**2AR and microglial cells. As well, recent studies targeting these receptors with long-acting agonists are considered with respect to their therapeutic potential in management of Parkinson**μ**s disease.

## 1. Introduction

Neurodegenerative disorders, such as Alzheimer's, Huntington's, and Parkinson's disease, are characterized by the progressive loss of the structure and function of neurons. Specifically, Parkinson's disease is characterized by the death of dopaminergic (DA) neurons (cell groups A8 and A9) in the midbrain, substantia nigra (SN), and striatum. Over time, this leads to impaired motor skills, shaking, slowness of movement, and postural instability, well known symptoms of PD. In addition, many patients experience dementia and executive dysfunction. A recent study of an unselected population-representative cohort (*n* = 142) highlights the poor prognosis of PD patients as evidenced by a 55% mortality rate within 10 years of diagnosis [1]. Of the survivors, 68% and 46% had postural instability and dementia, respectively; 23% had not yet progressed to either of these irreversible disease milestones.

Since the late 1960s, levodopa has been a key treatment and is the gold standard nearly 50 years later [2]. Converted to dopamine within the brain, it is used to control the motor symptoms of the disease but is ineffective with respect to dementia, freezing, or autonomic functions. A host of other treatments have been developed centered primarily around maintaining or increasing dopamine concentrations within the brain, such as dopamine agonists, MAO B inhibitors, catechol o-methyltransferase (COMT) inhibitors, anticholinergics, and amantadine. Stem cell transplant therapy and deep brain stimulation have also been explored with varying results.

What is known about neurodegenerative disorders in general and in Parkinson's disease in particular is that the progressive nature is in part associated with chronic inflammation and microglial activation [3]. Although a variety of triggers, including inherited genetic mutations and environmental toxins, can initiate the advent of neurodegeneration, inflammation is now recognized as an underlying mechanism that drives the progressive nature of Parkinson's disease [4–8]. As such, this review will examine the research exploring potential therapeutic targets aimed at abrogating the inflammatory dysfunction.

## 2. The Dysfunctional Immune System in Parkinson's Disease

Well over two decades ago, it was first recognized that activated microglia and neuroinflammation were associated with the SN lesions of PD patients when observed postmortem [8, 9]. Additionally, an accidental human neurotoxin model of Parkinson's disease also implicated activated microglia and chronic neuroinflammation in progressive loss of SN neurons [10, 11]. In this report, a group of drug users were exposed at relatively young ages to 1-methyl-4-phenyl-1,2,3,6-tetrahydropyridine (MPTP) as a contaminant in street-drugs. Each developed progressive Parkinsonian symptoms years after exposure. Postmortem analyses indicated the presence of neuroinflammation, activated microglia, and active neurodegeneration in the SN up to 15 years after systemic exposure to the MPTP contaminant [11]. Similar findings resulted from experiments with a long-term primate model of MPTP-induced SN degeneration [12]. These studies demonstrate that once a trigger initiates neuroinflammation in the SN, the condition can persist for long periods causing progressive loss of DA neurons.

Microglia are the innate immunity cells resident in the brain and are a major source of proinflammatory factors, such as tumor necrosis factor *α* (TNF*α*), interleukin-1 *β* (IL-1*β*), interleukin-6 (IL-6), reactive oxygen species (ROS), and nitric oxide as well as many others [3]. Microglia can also play beneficial roles in maintaining brain homeostasis and produce factors involved in neural cell survival, proliferation, growth, and motility. Microglia also secrete anti-inflammatory factors, including transforming growth factor *β* (TGF-*β*). As active immune sentinels, microglia have been observed *in vivo* to continually extend processes to probe their microenvironment even when in the so-called “resting” state [13]. However, after insult, microglia are rapidly activated, migrate to the site of injury, and phagocytose injured and dying cells [13].

**Figure 1:**
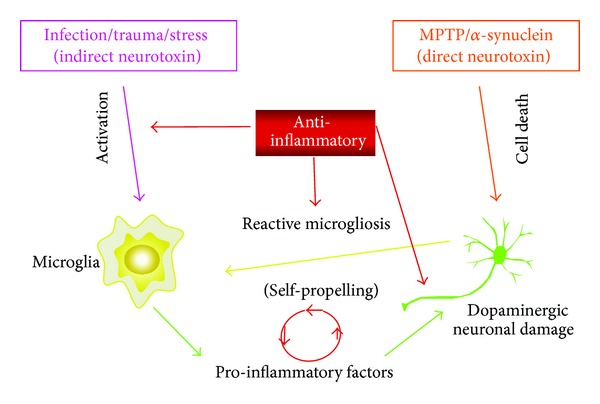
Model of neurodegeneration attributed to reactive microgliosis in Parkinson's disease.

In Parkinson's disease, it is these activated microglia that become self-propelling mediators of neuronal cell death leading to chronic inflammation. Once a trigger induces initial injury or death of DA neurons, activated microglia perpetuate the death of more neurons through the cyclic processes of pro-inflammatory reactive microgliosis [3, 14, 15]. In this manner, microglia mediate the progressive loss of DA neurons giving rise to characteristic postmortem PD reports of activated microglia, chronic inflammation, and loss of DA neurons within the SN. We have developed a model for the etiology of progressive Parkinson's disease (Figure 1). In this model, we believe that a direct neurotoxin, such as MPTP, or an inflammatory trigger, such as LPS, can lead to the direct or indirect activation of microglia. Animal models of neurodegeneration triggered by toxin exhibit similar progressive destruction of the SN.

## 3. ***β***2-Adrenergic Receptors

Microglia express high levels of *β*2AR at the cell surface [16]. Interestingly, several studies found that depletion of the endogenous *β*2AR agonist, norepinephrine (NE), caused increased microglial-induced neuroinflammation and that administration of NE protects cortical neurons from microglial-induced cell death [17, 18]. Furthermore, NE administration dose-dependently blocked microglial expression of the inflammatory mediators NOS2 and IL-1*β*, and these anti-inflammatory effects could be completely reversed by coapplication of the *β*2AR-specific antagonist, ICI-118,551 [18]. Together, these findings implicate *β*2AR as playing a key regulatory role in microglia activation.

Specifically, in humans the *β*2-adrenergic receptor (*β*2AR) is a 413 amino acid long glycoprotein that is a member of the seven-transmembrane (7TM) family of G-protein coupled receptors [19, 20]. Adrenergic receptors are subdivided into three groups (*β*1, *β*2, and *β*3) which are expressed in a variety of cell and tissue types, with the *β*2AR subtype classically occurring in the various cells of the pulmonary, cardiac, skeletal muscle, and immune systems [19]. Therapies that modulate *β*2AR responses are well documented for uses in treating asthma and other respiratory diseases, as well as hypertension and angina.

## 4. ***β***2AR Agonists

Beta-adrenergic receptor agonists are a group of drugs that are mimetics of endogenously occurring catecholamines, including epinephrine, norepinephrine, and dopamine. Agonists can be either direct interacting with the receptors or indirect in that they do not stimulate receptor activation but induce the release of endogenous catecholamines. There are multiple synthetic *β*2AR agonists which act mainly in the smooth muscle and endothelial cells of the pulmonary system, vasculature, bronchial tree, colon, and uterus. *β*2AR agonists have two functional forms: short-acting agonists used predominantly as fast-acting bronchodilators in treating asthma and other acute bronchial disorders, and long-acting agonists that are used to manage and control chronic, long-term bronchial diseases, such as *chronic obstructive pulmonary disease* (COPD). *β*2AR agonists have also been used to prevent premature labor by administration systemically to act upon the smooth muscle of the uterus.

### 4.1. Short-Acting versus Long-Acting *β*2AR Agonists

The molecular characteristics of the particular agonist determine the mode of interactions with *β*2AR and ultimately the manner in which downstream effects are generated. Agonists that have hydrophilic properties are able to access the receptor directly from the aqueous extracelluar environment. These agonists thus have relatively rapid effects and are generally termed “short-acting” due to the rapid onset and short duration of their stimulatory activity upon the receptor. Long-acting agonists are generally lipophilic and are taken up into the cell membrane where they slowly leach-out to access the *β*2AR over longer periods of time, thus producing more long lasting effects [19, 21]. Studies have shown that the *β*-ARs are also stereospecific and that this enantiomeric specificity can be important for agonist-induced functional responses from the stimulation of the receptor [22]. Furthermore, stereoselectivity by the receptor can determine the immunomodulatory effects of *β*2AR stimulation, especially with regards to the response of activated macrophages [19, 22]. However, studies have also shown in a model of cerebroischemic stroke that while two enantiomers of a lipophilic *β*2AR-agonist provide neuroprotection by activating astrocytes and inducing production of neurotropic factors, one racemate might also induce more adverse side-effects [23, 24].

## 5. ***β***2AR Agonists in Neuroprotection

Long-acting *β*2AR agonists that are typically used as bronchodilators in the treatment of asthma have also shown to have trophic effects in tissue culture models of CNS injury [25, 26]. Up-regulation of *β*2ARs after brain damage *in vivo* is associated with astrocyte activation and neuroprotection [27, 28]. Induction of neurotrophic growth factors and astrocyte activation by long-acting *β*2AR-agonists has been found to mediate neuroprotection in several *in vivo* models of neuronal damage [23, 29, 30]. *β*2ARs are also expressed in microglial cells and have been shown to mediate the inhibition of microglia activation [31]. However, *β*2AR *antagonists* are reported in another study to block IL-6 production and acute inflammatory response, as well as to provide neuroprotection in a model of hemorrhagic stroke [32]. In contrast, the same antagonist (butoxamine) did not have any neuroprotective effect in a model of focal cerebral ischemia [30]. In fact, in this model butoxamine actually abrogated the neuroprotection provided by treatment with *β*2AR agonists [30]. It is suggested that the different results for neuroprotection have more to do with the two different models of stroke brain-damage and the mechanisms responsible for the damage [33]. Additionally, in a rat model of traumatic brain injury (TBI), stimulation of *β*2AR with agonist decreased brain function impairment and improved recovery after injury [34]. This latter study found that the agonist stimulated neuroprotection after TBI correlated with decreased levels of blood glutamate levels which are typically elevated in response to injury and are generally neurotoxic [34]. However, a major caveat in interpreting all of these studies is that the models required pretreatment with the *β*2ARs agonists (or antagonist) in order for neuroprotection. In contrast, in an LPS-stimulated chronic model of Parkinson's disease, treatment with the long-acting agonist salmeterol after initiation of the disease prevented neurotoxicity via inhibition of reactive microglia [35].

## 6. ***β***2AR Agonists in the Treatment of Parkinson's Disease

In 2011, we looked at the effects of both the short-acting *β*2AR agonist, salbutamol, and a variety of long-acting *β*2AR agonists, including salmeterol, on the survival of DA neurons after induction of inflammation in different disease models of Parkinson's disease [35]. In mixed cell-cultures composed of primary mesencephalic neurons and glial cells that were treated with LPS, the long-acting agonists protected DA neurons from inflammation-induced cytotoxicity as did salbutamol. However, a short-acting agonist was able to confer neuro-cell protection only at much higher concentrations. In contrast to these beneficial effects in an inflammation-based model of neurotoxicity, we found that salmeterol treatment had less pronounced protective effects in a similar mixed cell model which featured induction of DA neuron death by MPTP. The MPTP metabolite MPP+ mediates direct toxicity on the neurons themselves and this neuronal death leads to the induction of reactive microgliosis, whereas LPS-induced neurotoxicity is mediated by the direct action of LPS on microglial cells leading to their production of inflammatory mediators, which leads to neuronal death and the continuing cycle of reactive microgliosis [3, 36]. However, when tested in two *in vivo* models of PD, the LPS-stimulated long-term mouse model and the acute MPTP model, salmeterol also exhibited some neuroprotective effects either by pretreatment (in the LPS-induced model) or by treatment with salmeterol post MPTP-injections respectively. These results suggested to us that long-acting *β*2AR agonists, such as salmeterol, might be developed for their anti-inflammatory effects to attenuate the progressive loss of DA neurons characteristic in Parkinson's disease and improve motor activity in patients. Additional studies using non-MPTP models of PD should help determine whether use of agonists such as salmeterol could prove beneficial in the management of Parkinson's disease.

## 7. ***β***2AR Agonists and Signaling Pathways

In addition to their role in stimulating G-protein coupled signaling, *β*2AR agonists also transduce signals from the receptor through its association with *β*-arrestins [37]. In addition to mediating receptor desensitization through blocking G-protein coupling with *β*2AR [38], *β*- arrestin can also link *β*2AR to the activation of other signaling pathways, such as the MAPK signaling cascade and the kinase complex that regulates activation/inhibition of the transcription factor, NF-*κ*B [37, 39]. Earlier studies found that the inflammatory effects of *β*2AR agonists at high concentrations were mediated through *β*2AR-induced cAMP production [40–42]. However, Qian and colleagues showed that, in primary murine-microglia cultures, the addition of *low* concentrations of salmeterol inhibited the LPS-induced production of inflammatory mediators, such as reactive oxygen species (ROS), TNF*α*, and nitric oxide (NO) [35]. This inhibition is *β*2AR and *β*-arrestin dependent but cAMP independent [35]. Thus, low doses of salmeterol might inhibit inflammation and promote neuroprotection by regulating receptor association with *β*-arrestins and *β*-arrestins-mediated function in other signaling pathways such as NF-*κ*B pathway and the MAPK signaling cascade.

### 7.1. Anti-Inflammation and NF-*κ*B

It has been shown that *β*2AR agonists, at low concentrations, stimulate anti-inflammatory effects by negative regulation of the transcription factor NF-*κ*B [39]. *β*-arrestin2 is a binding partner of I*κ*Ba which is an inhibitor of NF-*κ*B activation [39]. NF-*κ*B is a ubiquitously expressed transcription factor that regulates transcription of genes involved in immunity and inflammation [43]. Five members of the NF-*κ*B/Rel family of proteins, including p50, p52, p65 (RelA), c-Rel, and RelB, are expressed in mammalian cells. These NF-*κ*B proteins form various homo- and hetero-dimers in the cell cytosol where they are held in the inactive state by association with inhibitory proteins called I*κ*Bs [44]. A variety of signaling pathways converge at a kinase complex (NF-*κ*B-inducing kinase/NIK and I*κ*B kinase/IKK*α*-IKK*β*-IKK*γ*) that regulates NF-*κ*B activation by controlling the association with I*κ*Bs. When activated, the catalytic subunits of this kinase complex (IKK*α* and IKK*β*) phosphorylate I*κ*B*α* and target it for degradation. Degradation of I*κ*B*α* releases NF-*κ*B and unmasks the nuclear signal domain which targets NF-*κ*B translocation to the nucleus where it binds to the *κ*B site, and is functionally active in transcription of inflammatory genes. In this way, regulation of NF-*κ*B transcription activity depends upon interaction with I*κ*B, and in turn, proteins such as *β*-arrestin that bind the I*κ*B kinase complex and regulate I*κ*B are critical for regulating both I*κ*B and NF-*κ*B. Low-dose salmeterol has been shown to inhibit NF-*κ*B activation suppressing its translocation to the nucleus [35]. Thus the beneficial effects of salmeterol might be exerted at least in part through *β*-arrestin-mediated control of NF-*κ*B.

### 7.2. Anti-Inflammation and MAPK

Activation of *β*2AR can also stimulate the MAPK signaling cascade via a G-protein-independent but *β*-arrestin-dependent mechanism [45]. Previous studies also showed that activation of *β*2AR with high concentrations of salmeterol (10^−5^ M to 10^−6^ M) induced the MAPK signaling pathway leading to increased phosphorylation of ERK1/2 and resulting in proinflammatory effects in macrophages and primary microglia [46]. However, this proinflammatory effect is mediated through activation of the cAMP pathway, although it is PKA-independent. Of key interest is that these same high doses of salmeterol increased neurotoxicity in mixed cell cultures and that this was mediated through increased NADPH oxidase activity through an ERK-dependent pathway [47]. Conversely, low concentrations of salmeterol greatly reduced the LPS-stimulated phosphorylation/activation of components of the MAPK, namely, ERK1/2, p38, and JNK [35]. Thus, lower concentrations of the *β*2AR agonist apparently produce anti-inflammatory effects by inhibiting both MAPK cascade signaling and NF-*κ*B activation. Further supporting evidence is that low-dose salmeterol inhibits a common upstream effector for both MAPK and NF-*κ*B; TGF-beta activated kinase 1 (TAK1) has been shown to be a key regulatory component in various signaling pathways involved in immunity and inflammation [48]. Both MAPK and NF-*κ*B are downstream targets of TAK1 which is also the convergent effector for LPS/TRL- and TNF*α*-stimulated inflammation. Low doses of salmeterol (10^−9^ M and 10^−10^ M) can inhibit the activating phosphorylation of TAK1 in primary microglia. This suggests that the inhibitory effect on MAPK signaling and NF-*κ*B activation involves inhibition of TAK1. However, the link(s) between inhibition of TAK1 and the *β*-arrestin-dependent anti-inflammatory effects of low-dose salmeterol has yet to be determined.

### 7.3. Anti-Inflammatory Effects of Low-Dose Salmeterol

**Figure 2:**
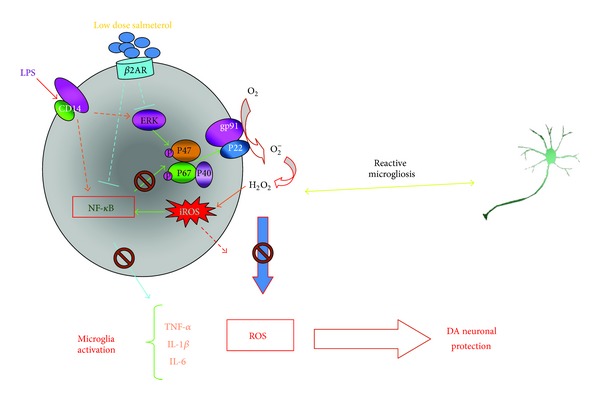
Molecular mechanism of the inhibitory function of salmeterol-mediated *β*2AR activation.

Previous results have shown that *β*2AR agonists are known to activate MAPKs via both Gs-dependent and Gs-independent mechanisms. A Gs-independent increase in phosphorylation of ERK occurres following high doses of salmeterol treatment in RAW264 macrophage cells and primary microglia cells [46], which mediates a proinflammatory and neurotoxic effect [47]. Conversely, much lower doses of salmeterol (10^−10^ − 10^−11^ M) have no proinflammatory effects but rather show dramatic inhibition of MAPK molecules ERK, JNK, and p38 in LPS-activated primary microglia. Although both effects appear to work independently of PKA activation, the proinflammatory effect of high-dose salmeterol is through the activation of the cAMP/EPAC pathway, and the inhibitory effect of low-dose salmeterol is independent of cAMP induction as well as PKA and EPAC activity. Rather, it appears as though the inhibitory activity of salmeterol is due to the activation of *β*-arrestin-2, which functionally inhibits both NF-*κ*B [39] and MAP-K [49]. In addition, low doses of salmeterol have a significant inhibitory effect on the LPS-mediated activation of NF-*κ*B and the production of inflammatory mediators normally under NF-*κ*B and MAP-K regulation, such as TNF-*α* and NO. However, this anti-inflammatory action of salmeterol may be selective only for certain proinflammatory pathways in microglial cells because low-dose salmeterol is able to inhibit the activation of superoxide production by LPS but not by PMA (which functions through the PKC pathway). Therefore, it appears that low-dose salmeterol can be potently but selectively anti-inflammatory in microglial cells by targeting the NF-*κ*B and MAPK signaling pathways following pro-inflammatory activation, as depicted in the model (Figure 2).

### 7.4. *β*-Arrestin-Biased Agonism

*β*-arrestin-biased agonism has been shown for a variety of *β*2AR agonists [37]. These agonists show preferential activation of noncanonical signaling that favors *β*-arrestin-mediated signals from the receptor over cAMP/PKA-mediated pathways. However, in the study by Drake and colleagues, salmeterol was not amongst these *β*-arrestin-biased agonists. Significantly, these studies used salmeterol at receptor-saturating *μ*M concentrations and showed that at these doses, salmeterol stimulated the production of cAMP and instigated cAMP-induced signaling [37]. Qian et al., in a 2009 study, found much the same results, that at high concentration, salmeterol increased the production of cAMP and downstream factors such NADPH oxidase activity [47]. These results are in sharp contrast to the effects of low concentrations of the same agonist, namely, that 10^−9^ to 10^−10^ M salmeterol does not stimulate increased cAMP and its downstream effectors but activates a *β*-arrestin-mediated reduction in some of these same downstream signaling events [35]. A distinction between the *β*-arrestin biased agonists and the nonbiased agonists reported by Drake et al. is the existence of a different structural characteristic between the two groups (the *β*-arrestin biased group all have an a-carbon ethyl substituent moiety lacking in the nonbiased group) [37]. It is interesting that Qian and colleagues essentially instigated the same *β*-arrestin-favored signaling by drastically reducing doses of salmeterol, a supposedly nonbiased *β*2AR agonist. Thus, *β*2AR agonists provide wide possibilities for utilizing biased signaling properties, from whatever mechanism of the bias, to develop therapeutic potential in novel disease backgrounds. In the case of inflammatory diseases such as Parkinson's disease, agonists, such as salmeterol, might be used therapeutically not only for their ability to inhibit inflammation and stop progressive neurotoxicity, but also to stimulate preferential signaling cascades that foster neurogenesis.

Salmeterol applied at concentrations from 1 nM to 100 nM has been shown to promote proliferation in adult rat dentate gyrus-derived neural precursor cells (ADP) [50]. Furthermore, it has also been found that neural progenitor cell proliferation is not necessarily cAMP-CREB-dependent [51]. Given that low doses of salmeterol preferentially stimulate *β*-arrestin signaling effectors as opposed to cAMP signaling events, the potential for neurogenic effects is intriguing at the least.

## 8. Concluding Remarks

The neuroprotective effects of *β*2AR activation by higher doses of *β*2AR agonists have been reported in other conditions, such as amyotrophic lateral sclerosis [52], cerebral ischemia [53], and spinal cord injury-induced locomotor dysfunction [54]. Although it is not yet clear how the *β*2AR agonists exhibit all of these neuroprotective properties, several studies have suggested that *β*2AR agonists function to stimulate glutathione-dependent antioxidant processes from nerve cells [30]. Meanwhile, others have reported that neurotrophic factors from activated astrocytes induced by *β*2AR agonists contribute to neuroprotection [55]. We propose that the major neuroprotective activity of *β*2AR agonists in Parkinson's disease models is due to their anti-inflammatory properties. It is clear that the effectiveness of the *β*2AR agonist, salmeterol, and the other long-acting *β*2AR agonists at low concentrations is due to their anti-inflammatory effect on microglia and not to a direct protective effect on DA-neurons or through an astrocyte-dependent effect. Given the effectiveness of these compounds at such low concentrations in inhibiting inflammatory responses, they appear to have significant potential in regulating CNS inflammation and the treatment of chronic inflammatory disorders of CNS.

## Abbreviations

*β*2AR: *β*2-adrenergic receptor
DA: Dopaminergic
IL: Interleukin
MPTP: 1-Methyl-4-phenyl-1,2,3,6-tetrahydropyridine
ROS: Reactive oxygen species
SN: Substantia nigra
TNF*α*: Tumor necrosis factor *α*.
